# Atrial Natriuretic Peptide and Renal Dopaminergic System: A Positive Friendly Relationship?

**DOI:** 10.1155/2014/710781

**Published:** 2014-06-12

**Authors:** Marcelo Roberto Choi, Natalia Lucía Rukavina Mikusic, Nicolás Martín Kouyoumdzian, María Cecilia Kravetz, Belisario Enrique Fernández

**Affiliations:** Department of Pathophysiology, Faculty of Pharmacy and Biochemistry, University of Buenos Aires, CONICET, INFIBIOC, 1113 Buenos Aires, Argentina

## Abstract

Sodium metabolism by the kidney is accomplished by an intricate interaction between signals from extrarenal and intrarenal sources and between antinatriuretic and natriuretic factors. Renal dopamine plays a central role in this interactive network. The natriuretic hormones, such as the atrial natriuretic peptide, mediate some of their effects by affecting the renal dopaminergic system. Renal dopaminergic tonus can be modulated at different steps of dopamine metabolism (synthesis, uptake, release, catabolism, and receptor sensitization) which can be regulated by the atrial natriuretic peptide. At tubular level, dopamine and atrial natriuretic peptide act together in a concerted manner to promote sodium excretion, especially through the overinhibition of Na^+^, K^+^-ATPase activity. In this way, different pathological scenarios where renal sodium excretion is dysregulated, as in nephrotic syndrome or hypertension, are associated with impaired action of renal dopamine and/or atrial natriuretic peptide, or as a result of impaired interaction between these two natriuretic systems. The aim of this review is to update and comment on the most recent evidences demonstrating how the renal dopaminergic system interacts with atrial natriuretic peptide to control renal physiology and blood pressure through different regulatory pathways.

## 1. Introduction

Renal sodium retention, a major determinant of hypertension, is regulated by a variety of endocrine, autocrine, and neuronal factors [1]. These factors regulate sodium metabolism by controlling the rate of sodium reabsorption at different tubular segments of the kidney [1]. According to Aperia A, we must consider the possibility that antinatriuretic as well as natriuretic factors may use common signaling pathways to increase or decrease natriuresis involving, respectively, the reversible activation or deactivation of the enzyme Na^+^, K^+^-ATPase in renal tubules [2].

Besides its well known role as a brain neurotransmitter, dopamine exerts specific functions at the periphery, with the most relevant effects being those on the cardiovascular system and the kidney [2, 3]. In 1964, it was reported that dopamine increases the glomerular filtration rate and promotes sodium excretion, and in 1972 the role of dopamine as a relevant autocrine and paracrine regulator of renal functions was reported for the first time [4, 5]. After that, several reports clearly demonstrated that the intrarenal synthetized dopamine constitutes a peripheral dopaminergic system and that renal dopamine is a modulator of blood pressure, sodium balance, and renal functions, independently of the neural dopaminergic system [3]. The importance of dopamine as a natriuretic hormone is reflected through its capacity to inhibit the majority of renal tubule sodium transporters [2, 6]. Notably, the activity of Na^+^, K^+^-ATPase is inhibited in most of the tubular segments by dopamine, where it acts by opposing the effects of antinatriuretic factors, such as angiotensin II (ANG II) [6, 7].

The atrial natriuretic peptide (ANP) discovered by de Bold et al. is a 28-amino acid peptide synthesized and stored in the atrial myocytes and released in response to the stretching of the cardiac wall or after stimulation with endothelin, citokines, or *α*-adrenergic agents [8–10]. ANP natriuretic effects are exerted by increasing the glomerular filtration rate and by inhibiting sodium tubular reabsorption [11]. In the proximal tubular cells, ANP inhibits ANG II sodium and water dependent reabsorption and also decreases water reabsorption by distal and collector tubules [12]. The inhibitory effects of ANP are mediated by a cyclic guanylate monophosphate- (cGMP-) protein kinase G- (PKG-) dependent mechanism [13].

## 2. Renal Dopamine: Function and Regulation Steps of a Local Natriuretic System

**Figure 1:**
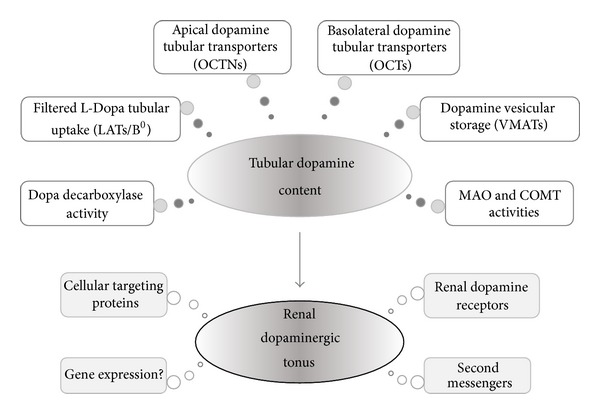
Major processes that regulate the endogenous content of dopamine in renal tubular cells and the renal dopaminergic tonus. B^0^: amino acid transport system B^0^; COMT: catechol-*O*-methyltransferase; LATs: L-type amino acid transporters; MAO: monoamine-oxidase; OCTs: organic cationic transporters; OCTNs: organic cation/carnitine transporters; VMATs: vesicular monoamine transporters.

Renal dopaminergic tonus can be modulated by the availability of intrarenal dopamine, which depends on the following: (a) the precursor L-Dopa that filters through the glomerulus and then is uptaken by the proximal tubules; (b) the synthesis of dopamine that is dependent on Dopa decarboxylase activity; (c) the release of dopamine from the tubular cell to the tubular fluid; (d) the uptake of dopamine from the circulation to the tubular cell; (e) the amount of dopamine storage into specific vesicles; (f) the activity of catechol-*O*-methyltransferase (COMT) and monoamine-oxidase (MAO), which are the catabolizing enzymes of dopamine (Figure 1) [14, 15].

Renal dopamine derives mainly from the local synthesis in the kidney. The dopamine precursor L-Dopa enters into the cells carried inward by sodium dependent and independent transporters [16]. After this, L-Dopa is converted into dopamine by Dopa decarboxylase, also named aromatic L-amino acid decarboxylase, which is present in high concentrations in the proximal tubular cells [17]. Unlike that in neural tissue, the dopamine synthesized by the renal tubules is not converted into norepinephrine [18]. The conversion of L-Dopa into dopamine in the kidneys provides virtually all the free urinary dopamine [19]. L-Dopa uptake by the proximal tubule cells is stimulated by Na^+^-independent and Na^+^-dependent transporters through the apical membrane [20]. The inward Na^+^ gradient apparently stimulates the apical Na^+^-dependent L-Dopa influx via a Na^+^-dependent L-Dopa transporter, namely, amino acid transport system B^0^ (system B^0^) [20–24]. This linkage between L-Dopa uptake and sodium transport explains why urinary dopamine increases after a high salt intake [20]. Therefore, L-Dopa uptake can be controlled by changes in salt intake [17]. In this way, it has been demonstrated that sodium retention leads to an increase in renal dopaminergic tonus, and the natriuretic effects of dopamine are more prominent under this condition. The inhibition or the downregulation of dopamine receptors significantly attenuates the natriuretic response to salt loading [2]. The Na^+^-independent mechanism involves other two transporters, namely, type 1 and type 2 L-amino acid transporters (LAT-1 and LAT-2, resp.), placed mainly on the lumen side of the proximal tubular cells [20]. L-Dopa supply to the proximal tubules increases dopamine local synthesis and consequently influences renal dopaminergic tonus. In agreement with this, it has been demonstrated that the acute administration of L-Dopa or *γ*-glutamyl L-Dopa (gludopa) increases the natriuresis, which is dopamine dependent, and simultaneously inhibits renal tubule Na^+^, K^+^-ATPase activity [25]. The inhibition of Dopa decarboxylase by carbidopa prevents the response of dopamine to sodium loading in both rats and humans and stimulates Na^+^, K^+^-ATPase activity [14, 26]. Dopa decarboxylase activity can be modulated by different regulatory factors. The enzyme can be upregulated by a high salt diet and downregulated by a low salt diet [27]. In this way, a salt loading to the kidneys results in an increase of Dopa decarboxylase activity in the proximal tubules [28]. Another fact to be considered is that urinary dopamine excretion, natriuresis, and diuresis are elevated in an animal model characterized by a moderate volume expansion, with these effects being reduced by the administration of a Dopa decarboxylase inhibitor (benserazide) [29]. Additionally, it has been demonstrated that the administration of carbidopa suppressed the basal Dopa decarboxylase-specific activity by 98% in renal cortex slices, suggesting that the decarboxylation activity belonged to Dopa decarboxylase and not to other decarboxylating enzymes [30]. This effect was associated with an increase of 56% in the Na^+^, K^+^-ATPase activity [31]. Similarly, ANG II reduces Dopa decarboxylase activity by 48% and under dopamine synthesis inhibition ANG II increases Na^+^, K^+^-ATPase activity by 35% [30, 31].

Besides L-Dopa transporters located in the proximal tubular cells, other nonneuronal transporters have been postulated as candidates for dopamine transport at the same location. Newly formed intracellular dopamine leaves the cell through the apical border by a diffusional process, whereas plasma dopamine can be uptaken through the basal cell border by a saturable process [32]. Both processes are, however, insensitive to the classical inhibitors of dopamine transporters, such as cocaine and GBR-12909 [32, 33]. Nonneuronal transporters can handle different exogenous and endogenous organic cations (with different affinities and kinetics) and still remain a subject of intense research up to date. Renal organic transporters are members of the group SLC22A (solute carrier superfamily), which includes the polyspecific organic cationic transporters: OCT-1, OCT-2, and OCT-3, located mainly at the basolateral membrane of proximal tubular cells, and OCTN-1, OCTN-2, and OCTN-3 located mainly at the apical side of the proximal tubular cells [32, 34, 35]. Among the catecholamines, dopamine is one of the endogenous organic cations to be uptaken or secreted by this kind of transporters [36]. A possible route for dopamine handling in the proximal tubules includes a two-step procedure: first, the electrogenic uptake of circulating dopamine at the basolateral membrane, mediated by OCTs, and second, the cation release at the luminal membrane, which is mediated by an electroneutral proton cation antiporter (OCTNs) [32, 37]. In this way, the OCT-2 appears to be the mainly basolateral transporter involved in the efflux of dopamine into the tubular lumen, since the intravenous administration of a potent inhibitor of OCT-2, Disprocynium 24, significantly reduces the tubular secretion of dopamine while it increases its spillover into the systemic circulation [38–40]. The physiological relevance of OCTs on dopamine handling in the kidney is linked to their regulation by protein kinase phosphorylations and by natriuretic and antinatriuretic hormones [41].

Aperia and coworkers were the first to publish in 1987 that renal synthetized dopamine, acting as a first messenger, was capable of regulating the activity of tubular Na^+^, K^+^-ATPase through a short term mechanism [42]. Dopamine inhibition of Na^+^, K^+^-ATPase and other tubular sodium transporters was then confirmed by different experimental studies [6]. Through this mechanism, most of the hormonal factors related to sodium excretion exert their natriuretic actions [2, 14, 31, 43]. These evidences permit concluding that renal sodium metabolism depends on an intact intrarenal dopaminergic system.

## 3. Regulation of Renal Dopamine System by the Atrial Natriuretic Peptide

Although renal dopamine can exert direct natriuretic effects by itself, it is possible that the amine can increase sodium excretion through indirect effects by adding its effects to other natriuretic hormones and opposing the antinatriuretic actions of other endogenous factors [31, 44]. Diverse mechanisms have been postulated to explain the putative interaction between renal dopamine and other hormones to regulate sodium excretion by the kidney [43, 44]. In this way, two major pathways have been involved. The first includes short-time and synergistic effects of renal dopamine, which can potentiate natriuretic ANP effects by one side or antagonize vasopressin actions or alpha adrenergic receptor stimulation by the other side [45–47]. The second pathway involves long-term effects, by which renal dopamine can increase sodium excretion by upregulating the synthesis of prostaglandins or by downregulating the expression of AT1 receptors [48, 49].

The observation that dopamine and ANP share some similar physiological effects suggests that each one may contribute by itself to enhance the actions of the other [50]. Diverse experimental studies have suggested a possible interaction between natriuretic peptide hormones and the renal dopaminergic system [15, 51, 52]. However, besides these reports, the mechanisms involved in ANP-dopamine interaction in the kidney remain unclear up to date and are still under study.

**Figure 2:**
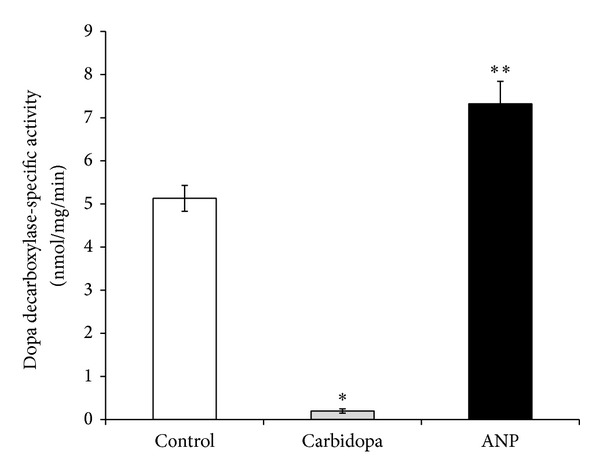
Effects of 200 *μ*M carbidopa and 100 nM ANP on dopamine formed as the product of Dopa decarboxylase-specific activity (nmol/mg of protein per minute ± SEM) on* in vitro* study using homogenates from rat renal cortex slices (*n*: number of samples); *P < 0.005* versus* control; **P < 0.05* versus* control. Control group: *n* = 7; carbidopa group: *n* = 6; ANP group: *n* = 6.

It has been reported that part of the inhibitory effects of ANP on sodium and water reabsorption is dependent on dopaminergic mechanisms, particularly those that involve the dopamine receptors [53–55]. Marin-Grez et al. and Webb et al. have reported that part of ANP inhibitory effects on sodium and water reabsorption is mediated by dopaminergic mechanisms, since haloperidol and the D_1_ receptor antagonist SCH 23390 partially blocked a significant percentage of the natriuretic and diuretic effects of ANP [53, 55]. In a similar fashion, ANP can modulate the metabolism of another catecholamine like noradrenaline, since it has been demonstrated that ANP increases norepinephrine uptake and its endogenous content and decreases its release, synthesis, and turnover and tyrosine hydroxylase activity at the hypothalamic presynaptic nerve ending level [56, 57]. The natriuretic peptides also regulate norepinephrine metabolism in the adrenal medulla [58]. Then, it is likely that ANP could regulate DA metabolism in the kidney as it does in the central nervous system and adrenal medulla. To support the idea that ANP may interact with renal dopamine to facilitate its actions, different experimental studies demonstrated that ANP enhances the inhibitory effect of dopamine on the Na^+^/H^+^ exchanger in the proximal tubules and also induces the recruitment of D_1_ receptors from the intracellular compartment to the plasma membrane, thereby facilitating their stimulation by dopamine [2, 59, 60]. Another study that complements and reinforces this idea showed that ANP stimulates dopamine uptake by the tubular cells in the kidney, via the stimulation of natriuretic peptide receptor type A (NPR-A) receptors coupled to guanylate cyclase, followed by cGMP (as second messenger) and protein kinase G (PKG) activation [17, 43]. This uptake process was characterized as a typical extraneuronal uptake and temperature dependent mechanism [43]. In addition, one* in vitro* study demonstrated that ANP increases Dopa decarboxylase activity by 42% in renal cortex slices (unpublished data) (Figure 2).

ANP also reduces COMT activity but lacks effect on renal dopamine release [52]. These findings altogether show that ANP contributes to increase endogenous dopamine content in the renal external cortex and points out that both systems interact to enhance their natriuretic and diuretic effects.

ANP and dopamine systems, through their second messengers and associated protein kinases or protein phosphatases, initiate a cascade of events ultimately resulting in the phosphorylation and inhibition of the enzymatic activity of Na^+^, K^+^-ATPase [2, 17, 43].

**Figure 3:**
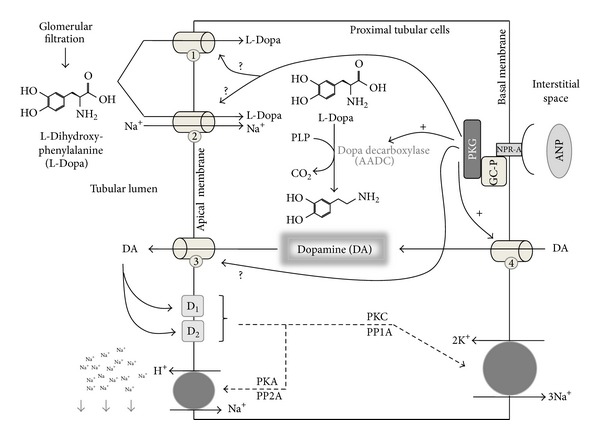
The synthesis and tubular handling of dopamine. Filtered L-Dopa can be uptaken by two different mechanisms: (1) sodium independent transporters (LATs) and (2) sodium dependent transporters (system B^0^). L-Dopa is rapidly decarboxylated to dopamine (DA) by Dopa decarboxylase enzyme, using pyridoxal 5-phosphate hydrate (PLP) as cofactor. Dopa decarboxylase activity can be regulated by different hormones, like angiotensin II or atrial natriuretic peptide (ANP), among others. Newly formed dopamine can leave the cells through the apical border (3), probably mediated by organic cationic transporters (OCTNs). Circulating dopamine can enter into proximal tubular cells through basal located OCTs (4). Full arrows and “+”: stimulating action of PKG; full arrows and question mark “?” suggest a possible regulatory mechanism; dashed arrows suggest the intracellular signaling.

In this way, ANP favors dopamine intracellular accumulation, which in turn permits D_1_ receptors recruitment and stimulation, resulting in the overinhibition of Na^+^, K^+^-ATPase activity, the decrease of sodium reabsorption, and the increase of natriuresis [17]. In agreement with this, it has been demonstrated that, under dopamine synthesis inhibition, dopamine and ANP added simultaneously significantly decrease Na^+^, K^+^-ATPase activity by 50% with respect to dopamine or ANP alone [17]. Moreover, the addition of hydrocortisone (an extraneuronal dopamine transporter inhibitor) reversed ANP-dopamine overinhibition of the enzyme, demonstrating that ANP enhances dopamine uptake through tubular transporters (Figure 3) [17]. Thus, dopamine and ANP may achieve their effects through a common pathway that involves reversible deactivation of renal tubular Na^+^, K^+^-ATPase activity [17, 43]. The signaling pathway mechanism by which these two hormones enhance their natriuretic action could involve the dopamine and cAMP-regulated phosphoprotein (DARPP-32). The knockout of this intracellular messenger in mice leads to a hypertensive state where ANP cannot exert its natriuretic action [61]. It must be pointed out that PKG and PKC, activated by ANP and dopamine, respectively, stimulate DARPP-32 phosphorylation, which in turn induces the inactivation of Na^+^, K^+^-ATPase [17, 62]. This suggests that DARPP-32 may also be involved in the modulation and interaction of ANP and dopamine renal systems [61, 62].

Controversially, other authors suggested that there is no relationship between ANP and dopamine systems [63]. Murphy and Bass reported that it is unlikely that dopamine generation or release, or dopamine receptor activation (with the possible exception of its involvement in the rat), is essential to express ANP effects. They postulate that dopamine actions do not depend on ANP release and that there are no studies about dopamine activation of ANP receptors [63]. On the other hand, Hirata et al. suggested that the renal effects of ANP and dopamine utilize different pathways, since ANP and DA effects on both glomerular filtration rate and sodium urinary excretion are additive. They also reported that, in contrast to dopamine, ANP increased arterioles efferent resistance and nephrogenous cGMP [64]. A complementary study carried out in humans demonstrated that the infusion of a low dose of ANP (1.5 pmol/kg/min) did not produce hemodynamic or natriuretic effects, but a higher dose (15 pmol/kg/min) increased urinary sodium excretion, but is lacking hemodynamic actions. In addition, the administration of both doses of ANP did not alter urinary dopamine excretion [65].

The described interaction between circulating ANP and locally formed dopamine should contribute to the maintenance of a well-balanced regulation of sodium metabolism and blood pressure. The impairment of this relationship may also play a relevant role in the pathophysiology of experimental and human hypertension. In this way, the possibility that altered uptake of dopamine by renal tubules could be contributing to the development of essential hypertension might be considered.

## 4. Atrial Natriuretic Peptide and Dopamine Interaction: Pathophysiological Implications

Sodium retention is one of the main features of hypertension [66]. Renal dopamine is critical for the preservation of a normal hydroelectrolyte balance, a steady redox state, and normal blood pressure levels [18]. Experimental and clinical studies indicate that the impairment of the renal dopaminergic system plays a major role in the pathogenesis of different types of hypertension [67–69]. On the other hand, renal dopamine receptors can modulate the production of reactive oxygen species (ROS), by interacting with the renin-angiotensin and sympathetic nervous systems [3]. In the last years, several experimental studies demonstrated the role of oxidative stress and its association with the impairment of dopamine receptor functions in the pathophysiology of hypertension [68, 70–72]. Hypertension can be associated with the absence of any of the five dopamine receptor subtypes [68, 70, 71]. Dopamine, through stimulation of D_1_, D_2_, and D_5_ receptors, regulates the redox balance and exerts antioxidant effects by direct and indirect inhibition of prooxidant enzymes, such as nicotinamide adenine dinucleotide phosphate reduced form (NADPH) oxidase, and stimulation of antioxidant enzymes, which in turn may also indirectly inhibit NADPH oxidase activity [68, 70]. In this way, activation of dopamine D_2_ receptor inhibits NADPH oxidase activity by increasing the expression of endogenous antioxidants, like Parkinson protein 7 (PARK7 or DJ-1), paraoxonase 2 (PON2), and heme oxygenase 2 (HO-2) [68]. Furthermore, stimulation of the D_5_ receptor decreases NADPH oxidase activity by inhibition of phospholipase D_2_ and increases the expression of the antioxidant HO-1 [68]. Meanwhile, the D_1_ receptor inhibits NADPH oxidase activity through stimulation of the protein kinase A-protein kinase C cross-talk [70, 71]. Another study demonstrated that oxidative stress via nuclear factor kappa-light-chain-enhancer of activated B cells (NF-*κ*B) activation downregulates D_1_ receptor function causing a decrease in sodium excretion, which contributed to an increase in blood pressure [72]. This was confirmed by the fact that inhibition of oxidative stress and NF-*κ*B activation through stimulation of a redox-sensitive transcription factor (nuclear factor E_2_-related factor 2- (Nrf2-) phase II antioxidant enzyme pathway) by sulforaphane maintains D_1_ receptor functionality and prevents the development of hypertension [72]. Atrial natriuretic peptide, produced and released by myocytes in response to high blood pressure, can suppress renin-angiotensin system and reduce water and sodium loads on the circulatory system to diminish blood pressure levels [73]. Several studies indicate that, in addition to these effects, ANP can act to preserve renal function [74, 75]. Koga et al. showed in rats that ANP also has antioxidant effects as demonstrated by its ability to attenuate ROS levels in a renal ischemia-reperfusion injury model [76]. In another study using uremic rats by 5/6 nephrectomy, progression of renal deterioration and renal oxidative stress was accompanied with an increase of ANP mRNA 133-fold in tandem with the increase in blood pressure, suggesting that ANP mRNA may be increased as a protective mechanism [73]. Taking this evidence into consideration, renoprotective effects of ANP and dopamine combination must be further investigated.

The proteinuria (>3.5 g/day) and sodium retention observed in nephrotic syndrome conduce to the development and maintenance of edema and ascites [77]. Besides hypovolemia and hypoalbuminemia, a primary abnormality in intrarenal sodium handling is also implicated in the nephrotic syndrome [77, 78]. It has been demonstrated that this impairment of sodium excretion in the nephrotic syndrome is due to an increase in the Na^+^/H^+^ exchanger (NHE3) activity in the proximal tubules, together with a shift of NHE3 from an inactive to an active form [79]. Furthermore, other authors have attributed this disturbance to a blunted response to ANP and also to an increase of Na^+^, K^+^-ATPase activity at the cortical collecting duct level [80, 81]. In addition, Sampaio-Maia and coworkers showed a decrease in renal Dopa decarboxylase activity with lower urinary excretion of dopamine and a reduced availability of D_1_ receptors in proximal tubules as a sign of impairment of the renal dopaminergic system in rats with puromycin aminonucleoside- (PAN-) nephrotic syndrome [82]. The participation of D_1_ receptors as a contributor factor of sodium retention was supported by the fact that the infusion of the D_1_ receptor agonist fenoldopam was not able to enhance the natriuretic response in PAN-nephrotic syndrome rats or reverse Na^+^, K^+^-ATPase increased activity under normal or volume expanded conditions [82]. Atrial natriuretic peptide and intrarenal dopamine interact with each other in the regulation of sodium homeostasis [51]. It is noteworthy that, between other mechanisms of interaction, ANP stimulates the recruitment and activation of silent D_1_ receptors from the cytosolic compartment towards the plasma membrane of renal tubular cells [83]. The impairment of ANP-dopamine interaction also contributes to sodium retention in the experimental PAN-nephrotic syndrome [51]. In this model, the unresponsiveness of ANP receptors and the decrease of D_1_ receptors expression in renal tubules have been reported [51]. Although circulating ANP levels in PAN-nephrotic syndrome are elevated, the increase of natriuresis and urinary cGMP excretion evoked by an acute volume expansion were blunted [51]. The fact that the infusion of the phosphodiesterase type 5 inhibitor, zaprinast, restored D_1_ receptors in tubular cells, cGMP urinary excretion, and natriuresis to control levels supports the hypothesis of a dysfunction in ANP and D_1_ receptors in nephrotic syndrome [51]. The authors concluded that D_1_ receptors may play a major role in ANP resistance observed in PAN-nephrotic syndrome, since coadministration of SCH-23390 (a D_1_ receptor antagonist) abolished the effects elicited by zaprinast [51].

## 5. Future Perspectives

Appropriate regulation of renal dopaminergic tonus is one important requisite for the maintenance of sodium homeostasis and normal blood pressure. Identification of abnormalities in different steps of crucial importance for the regulation of the renal dopaminergic tonus should provide new tools for early detection of individuals that are predisposed to develop hypertension. Recently, it has been demonstrated that renal gastrin and dopamine receptors interact to synergistically increase sodium excretion and that the impairment of this interaction may be involved in the pathogenesis of hypertension [84]. Atrial natriuretic peptide, which targets D_1_ receptors at the plasma membrane and appears to have many of its renal effects mediated via the D_1_ receptor, could open up new therapeutic possibilities.

Natriuretic peptides may exert beneficial effects on nephrogenic diseases characterized by sodium and water retention. In this way the clinical use of synthesized analogs of natriuretic peptides such as anaritide, vasonatrin, ularitide, and nesiritide must be considered in the future [85–87]. Furthermore, pharmacological agents with dual action (inhibitor/enhancer) on the renin-angiotensin and natriuretic peptide systems like omapatrilat have been used successfully in clinical trials for hypertension treatment [88, 89].

Another fact is that the renal dopaminergic system is sensitized by high salt intake and volume expansion, which raises the question about how intrarenal sodium sensors influence the bioavailability of renal dopamine. This approach may lead to the development of new pharmacological strategies in conditions of salt retention and hypertension. In this way, it is important that drugs developed to enhance the renal dopamine tonus are mainly targeting the kidney.

The recent awareness of the fact that dopamine has beneficial renal effects that are opposed to oxidative stress and inflammatory renal damage enables its use as a renoprotective agent in the future. It is mandatory to carry out clinical studies to demonstrate the participation of renal dopaminergic system in pathological contexts involving impaired sodium excretion as nephrotic syndrome or insulin resistance states. Finally, novel physiological functions have been discovered in recent years for natriuretic peptides, such as activation of lipolysis, lipid oxidation, and mitochondrial respiration [90]. This could open a possible therapeutic intervention against pandemic diseases such as obesity and insulin resistance.

## 6. Conclusions

Dopamine and ANP are involved in the management of renal sodium excretion and in the pathophysiology of different kinds of experimental as well as human hypertension. Current evidence suggests that there is a synergism between the renal dopaminergic system and ANP to increase sodium excretion. Atrial natriuretic peptide interacts with renal dopaminergic system reinforcing its natriuretic and diuretic effects through different mechanisms that favors dopamine intracellular accumulation. The increment of endogenous DA inside tubular cells would permit D_1_ receptors recruitment and stimulation and, in turn, overinhibition of Na^+^, K^+^-ATPase activity. The possibility that impaired ANP-dopamine interaction by renal tubules may play a role in the pathogenesis of hypertension should be considered.

Sodium retention in nephrotic syndrome is attributed to a blunted response to ANP and enhanced Na^+^, K^+^-ATPase activity in the cortical collecting duct, suggesting that dysfunction of renal dopaminergic and natriuretic peptide systems contributes to sodium retention in the experimental nephrotic syndrome. Both dopamine and ANP have protective effects on oxidative stress in the kidney. However, the ultimate clinical importance of these effects remains to be determined.
